# Preclinical Detection of Alpha-Synuclein Seeding Activity in the Colon of a Transgenic Mouse Model of Synucleinopathy by RT-QuIC

**DOI:** 10.3390/v13050759

**Published:** 2021-04-26

**Authors:** Jung-Youn Han, Chaewon Shin, Young Pyo Choi

**Affiliations:** 1 Laboratory Animal Center, Division of Research Strategy, Korea Brain Research Institute, 61 Cheomdan-ro, Dong-gu, Daegu 41068, Korea; iiswear@kbri.re.kr; 2 Department of Neurology, College of Medicine, Chungnam National University, Daejon 35015, Korea; chroma0202@gmail.com

**Keywords:** RT-QuIC, alpha-synuclein, synucleinopathy, Parkinson’s disease

## Abstract

In synucleinopathies such as Parkinson’s disease (PD) and dementia with Lewy body (DLB), pathological alpha-synuclein (α-syn) aggregates are found in the gastrointestinal (GI) tract as well as in the brain. In this study, using real-time quaking-induced conversion (RT-QuIC), we investigated the presence of α-syn seeding activity in the brain and colon tissue of G2-3 transgenic mice expressing human A53T α-syn. Here we show that pathological α-syn aggregates with seeding activity were present in the colon of G2-3 mice as early as 3 months old, which is in the presymptomatic stage prior to the observation of any neurological abnormalities. In contrast, α-syn seeding activity was not detectable in 3 month-old mouse brains and only identified at 6 months of age in one of three mice. In the symptomatic stage of 12 months of age, RT-QuIC seeding activity was consistently detectable in both the brain and colon of G2-3 mice. Our results indicate that the RT-QuIC assay can presymptomatically detect pathological α-syn aggregates in the colon of G2-3 mice several months prior to their detection in brain tissue.

## 1. Introduction

Alpha-synuclein (α-syn) is a neuronal protein of 140 amino acids predominantly localized to presynaptic nerve terminals [1,2]. α-synucleinopathies (or synucleinopathies) are a group of neurodegenerative diseases characterized by the accumulation of pathological α-syn aggregates in the brain [3,4]. The three major phenotypes include Parkinson’s disease (PD), dementia with Lewy bodies (DLB) and multiple system atrophy (MSA), in addition to several rarer diseases such as pure autonomic failure (PAF) [5,6]. While α-syn deposits appear in neuronal soma and processes (known as Lewy bodies and Lewy neurites, respectively) in PD and DLB, α-syn-positive glial cytoplasmic inclusions in oligodendrocytes are the neuropathological hallmark of MSA [4,7,8,9]. In patients affected by the various synucleinopathies, pathological α-syn deposits develop not only in the central nervous system (CNS) but also in the peripheral nervous system (PNS) of multiple organs, including stellate and sympathetic ganglia, the gastrointestinal (GI) tract, the heart and the adrenal gland [10,11,12,13]. Moreover, α-syn deposits in the enteric nervous system (ENS) of the GI tract from PD patients were reported to develop up to 20 years prior to the onset of motor symptoms [14]. Nonetheless, a number of immunohistochemistry (IHC)-based studies aiming to detect pathological α-syn inclusions in GI biopsies of PD patients have yielded conflicting results regarding the diagnostic accuracy of this approach [15,16,17,18,19,20]. The reported discrepancies between studies that examined GI biopsies of PD patients might be associated with variable amounts of pathological α-syn aggregates in the examined samples, methodological variation in IHC and the potentially variable expertise in the interpretation of results [21,22,23,24].

Real-time quaking-induced conversion (RT-QuIC) is a seeded cyclic amplification technique developed to detect very small amounts of disease-specific prion protein aggregates (PrP^Sc^) with high sensitivity and specificity [25,26]. Due to its ability to detect femtogram (10^−15^) levels of PrP^Sc^ [27], RT-QuIC has been extensively investigated during the last decade for diagnostic applications using cerebrospinal fluid (CSF) or other tissue outside the brain [28,29,30,31,32,33,34,35,36]. Recently, based on the prion-like properties of misfolded α-syn aggregates [37,38,39,40], RT-QuIC was successfully adapted to synucleinopathies [41,42]. Subsequently, a number of studies employing this assay successfully detected α-syn seeding activity with high sensitivity and specificity in CSF or peripheral tissues from patients affected by a variety of synucleinopathies [43,44,45,46,47,48,49].

In this study, using the α-syn RT-QuIC assay that we reported recently [50], we comparatively investigated the presence of α-syn seeding activity in the brain and colon of transgenic mice expressing an A53T mutant form of human α-syn. Our results show that α-syn seeding activity occurs in the colon of presymptomatic mice, and precedes the appearance of seeding activity in the brain.

## 2. Materials and Methods

Transgenic mice overexpressing human α-syn with an A53T mutation under the control of mouse prion protein promoter (line G2-3) was described previously [51]. The G2-3 mice develop neurological disease at ~12 months of age, which is characterized by rapidly progressive motor dysfunction leading to death within a few weeks after the clinical onset [51,52]. The mice display a neuropathological phenotype including loss of motor neurons and neuronal accumulation of pathological α-syn aggregates [51,53]. Ethical approval for the use of mice was obtained from the Institutional Animal Care and Use Committee of Korea Brain Research Institute (KBRI). Mice transgenic for human A53T α-syn and non-carrier controls were euthanized at 1, 3, 6 and 12 months of age. At each timepoint, brain and colon tissue samples were recovered. Subsequently, a caudal part of the brain (brainstem and midbrain) and a distal portion of the colon were separated and then homogenized in phosphate-buffered saline (PBS, pH 7.4) containing complete EDTA-free protease inhibitors and phosphatase inhibitors (Roche Applied Science, Upper Bavaria, Germany), by using the Precellys 24 tissue homogenizer (Bertin Instrument, Frankfurt, Germany). The 5% (*w*/*v*; colon) or 10% (*w*/*v*; brain) homogenates were clarified by centrifugation at 2000× *g* for 2 min and the supernatants were kept in aliquots at −80 °C until needed.

RT-QuIC analysis for brain or colon homogenates was performed as described previously [41] with some modifications [42]. Recombinant full-length human α-syn protein (140 amino acids) carrying an A53T mutation (rPeptide) was used as substrate. With regard to seeds, tissue homogenates kept at −80 °C were thawed on the day of analysis and sonicated as described [50]. Sonicated tissue homogenates were serially diluted and then used as seeds at indicated dilutions. Dilutions were expressed in relation to brain or colon tissue; for example, 10^−2^ dilution is equivalent to 1% tissue homogenate. A 96-well black plate with clear bottom (Nalgene Nunc.) was used for this assay. Each well of the plate was preloaded with 4 glass beads (1.0~1.25 mm in diameter) before the addition of 100 μL reaction mixture containing 10 μM recombinant α-syn, 10 μM thioflavin T (ThT) and 2 μL seeds in 100 mM phosphate buffer (pH 8.2). Individual samples were prepared in quadruplicate. The plate was sealed and then incubated at 37 °C with alternating 1 min shake and 1 min rest cycles (400 rpm, double orbital) in a FLUOstar Omega plate reader (BMG Labtech, Ortenberg, Germany). The plate reader measures ThT fluorescence in relative fluorescence units (RFU) and is saturated at 260,000 RFU. ThT fluorescence was measured at the starting point of the assay and then at one-hour intervals from the bottom of the wells (440 nm excitation and 480 nm emission, gain of 1750, 20 flashes per well). Fluorescence values at each measurement were shown as average for quadruplicate wells on the graph. A ThT fluorescence threshold in each plate was determined by averaging the fluorescence of the first five measurements for all samples plus 10 standard deviations (SD). Samples were considered positive when at least two of four replicates crossed this threshold. RFU_max_/RFU_iniital_ ratios were calculated by dividing maximal ThT fluorescence over the 40-h reaction (RFU_max_) by ThT fluorescence at the starting point (RFU_initial_).

## 3. Results

**Figure 1:**
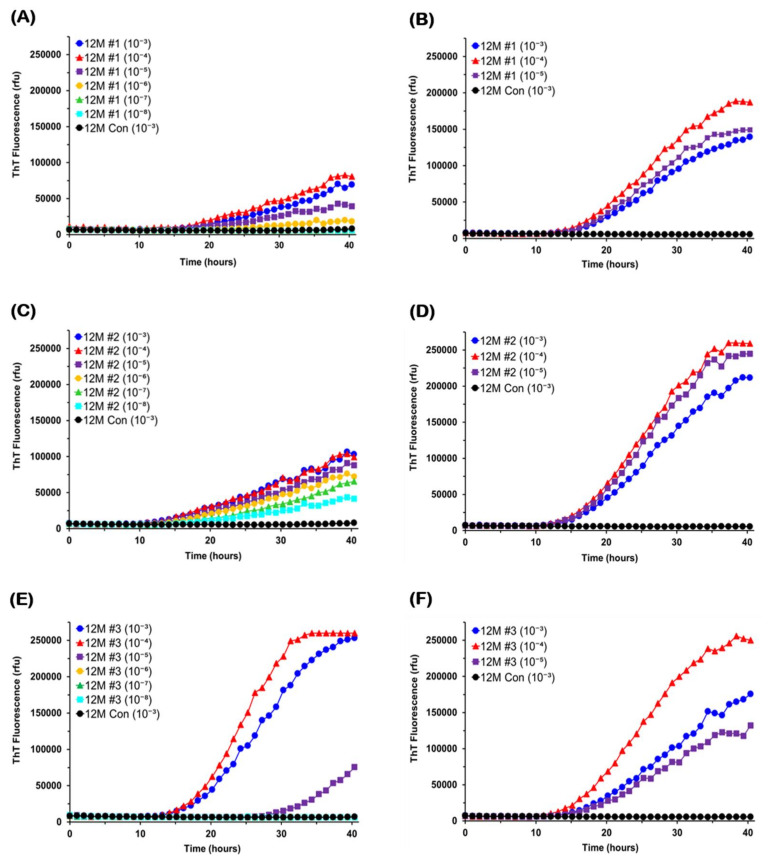
**RT-QuIC detection of α-syn seeding activity in both the brain and colon of 12-month-old G2-3 mice.** Serial dilutions of brain homogenates (10^−3^ to 10^−8^) or colon homogenates (10^−3^ to 10^−5^) from 12-month-old G2-3 mice were used to seed RT-QuIC reactions with A53T recombinant α-syn as substrate. RT-QuIC responses to brain samples were shown in the left column (**A**,**C**,**E**) and those to colon samples were shown in the right column (**B**,**D**,**F**). As controls, reactions were also seeded with 10^−3^ dilution of brain tissue or colon tissue from a non-carrier mouse of the same age. ThT fluorescence was measured at one-hour intervals and average values from quadruplicate wells were plotted as a function of time.

Given that G2-3 mice are known to develop rapidly progressive neurological symptoms at ~12 months, we first investigated α-syn seeding activity in brain and colon tissues of 12-month-old mice using RT-QuIC. Positive RT-QuIC responses were seen in all samples recovered from 12-month-old mice. For brains, α-syn seeding activity was identified up to 10^−5^ dilution in two of three mice (Figure 1A,E) or up to 10^−8^ dilution in the remaining mouse (Figure 1C). In colon tissue, RT-QuIC seeding activity was detectable up to 10^−5^ dilution in all three mice (Figure 1B,D,F). Further dilutions of colon tissue samples did not show any positive response in all three mice and were not investigated further (data not shown). While the two tissue types in 12M-#1 and #3 mice showed similar levels of α-syn seeding activity, its level in the brain of 12M-#2 mouse was 10^3^ times higher than in the colon (Table 1). Responses rising above the threshold were usually observed after 14–20 h in reactions seeded with 10^−3^ to 10^−5^ dilutions of both tissue homogenates, except for the brain of 12M-#1 mouse that required 22–27 h of lag phase to cross the threshold at indicated dilutions (Figure 1A). There were longer lag phases up to 26 h in reactions seeded with 10^−6^ to 10^−8^ dilutions of brain tissue from 12M-#2 mouse (Figure 1C). We observed that reactions seeded with the 10^−3^ dilution of tissue homogenates often gave weaker responses with longer lag phases and lower RFU_max_/RFU_iniital_ ratios than those seeded with 10^−4^ or even higher dilutions, which were more prominent in colon samples (Figure 1 and data not shown). This observation is thought to be associated with the presence of inhibitory components in tested samples that interfere with the RT-QuIC reaction [26,54]. It appears that 10^−3^ dilution was not enough to fully reduce the reaction inhibitors to subinhibitory concentrations in some samples, particularly colon samples, and further dilution to 10^−4^ is required for full removal of inhibitors as described previously [55].

**Figure 2:**
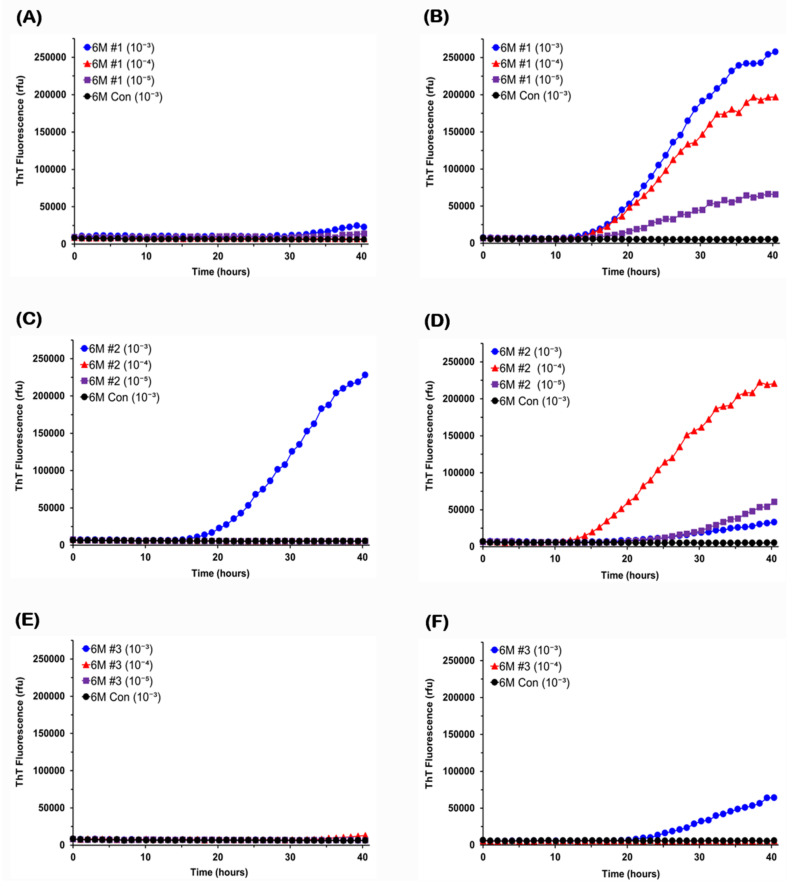
**RT-QuIC analysis of brain and colon samples from 6-month-old G2-3 mice.** Serial dilutions of brain or colon homogenates (10^−3^ to 10^−5^) from 6-month-old G2-3 mice were used to seed RT-QuIC reactions with A53T recombinant α-syn as substrate. RT-QuIC responses to brain samples were shown in the left column (**A**,**C**,**E**) and those to colon samples were shown in the right column (**B**,**D**,**F**). As controls, reactions were also seeded with 10^−3^ dilution of brain tissue or colon tissue from a non-carrier mouse of the same age. ThT fluorescence was measured at one-hour intervals and average values from quadruplicate wells were plotted as a function of time.

We then investigated brains and colons of 6-month-old mice using RT-QuIC, given previous studies in which pathological α-syn was not yet detectable in the brain of G2-3 mouse at this age but appeared at 7–8 months of age [56]. As shown in Figure 2A,E, α-syn seeding activity was not detectable in the brains of two 6-month-old mice. Positive responses were seen only in reactions seeded with 10^−3^ dilution of the brain tissue from the 6M-#2 mouse (Figure 2C). In contrast to the brain, colon samples from all three 6-month-old mice had detectable seeding activity at 10^−3^ dilution (Figure 2F), and even up to 10^−5^ dilution (Figure 2B,D). Therefore, α-syn seeding activity occurred first in the colon at least in two of the three 6-month-old mice, and its colon levels were higher than in the brains of all three mice (Table 1). The lag times varied between samples and degrees of dilution, ranging from 15 to 30 h.

**Figure 3:**
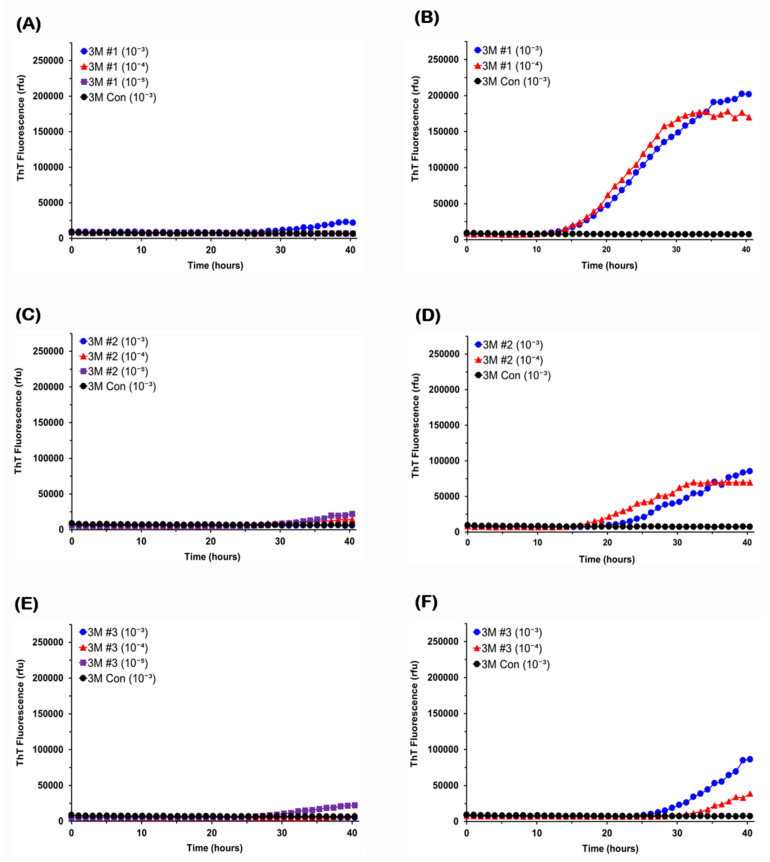
**Alpha-synuclein seeding activity is present in the colon but absent in the brain of 3-month-old G2-3 mice.** Serial dilutions of brain homogenates (10^−3^ to 10^−5^) or colon homogenates (10^−3^ to 10^−4^) from 3-month-old G2-3 mice were used to seed RT-QuIC reactions with A53T recombinant α-syn as substrate. RT-QuIC responses to brain samples were shown in the left column (**A**,**C**,**E**) and those to colon samples were shown in the right column (**B**,**D**,**F**). As controls, reactions were also seeded with 10^−3^ dilution of brain tissue or colon tissue from a non-carrier mouse of the same age. ThT fluorescence was measured at one-hour intervals and average values from quadruplicate wells were plotted as a function of time.

**Figure 4:**
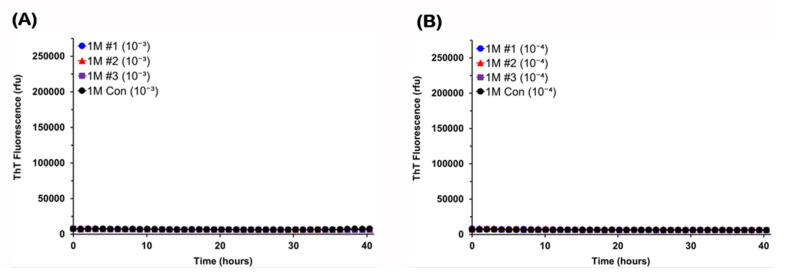
**Alpha-synuclein seeding activity is absent in the colon of 1-month-old G2-3 mice.** Colon homogenates were prepared from three G2-3 mice of 1 month of age and one non-carrier mouse of the same age. Colon homogenates at 10^−3^ dilution (**A**) or 10^−4^ dilution (**B**) were used to seed RT-QuIC reactions with A53T recombinant α-syn as substrate. ThT fluorescence was measured at one-hour intervals and average values from quadruplicate wells were plotted as a function of time.

Next, considering that GI deficits similar to those occurring in the prodromal phase of PD were reported to develop at 3 months of age [57], brains and colons recovered from G2-3 mice of this age were examined for α-syn seeding activity. As shown in Figure 3 (left column), all reactions seeded with 10^−3^ to 10^−5^ dilutions of brain tissue from 3-month-old mice gave flat responses in the assay. In contrast, colon tissue from all three 3-month-old mice contained α-syn seeding activity detectable only at 10^−3^ dilution (3M-#2 and -#3) or up to 10^−4^ dilution (3M-#1) (Figure 3, right column). Further dilutions of colon tissue of 3M-#1 mouse did not show any positive response (data not shown). The lag phases varied from 18 to 34 h between colon samples. Positive responses at 10^−3^ dilution of colon tissue of 3M-#2 or 3M-#3 mouse were observed only in partial wells (two or three of four), indicating low seeding doses in those samples that were barely detectable in this assay (Figure 3D,F). Additionally, reactions seeded with 10^−4^ dilution of colon tissue of these two mice were determined to be negative, since only one of four wells gave responses rising above the threshold (Figure 3D,F; Table 1). We occasionally observed such a spontaneous positive response, as described previously [50]. Lastly, colon tissue recovered from 1-month-old G2-3 mice were examined for α-syn seeding activity. All reactions seeded with 10^−3^ or 10^−4^ dilution of colon tissue from 1-month-old mice gave flat responses in the assay (Figure 4), indicating the absence of α-syn seeding activity in the colon of G2-3 mice at this age.

## 4. Discussion

In this study, we show that our RT-QuIC assay was able to detect pathological α-syn aggregates with seeding activity in the colon of 3-month-old G2-3 mice, which is in the preclinical stage prior to any motor impairments or CNS pathology [51]. In contrast, α-syn seeding activity was not detectable in the brain at 3 months of age and appeared at 6 months of age only in one of three mice. At 12 months, which is in the clinical stage presenting with neurological abnormalities [51,53,56], RT-QuIC seeding activity was found in both types of tissue in all three mice. Collectively, our results clearly demonstrate that α-syn seeding activity in the colon precedes its occurrence in the brain of this transgenic mouse model of synucleinopathy. In agreement with our results, GI dysfunction including constipation and α-syn deposits in enteric neurons was described in the G2-3 mice from 3 months of age [57]. Therefore, given the presence of GI abnormalities for several months prior to the emergence of pathological changes in the brain, this G2-3 line can be a useful model in developing treatments to stop or delay disease progression during the prodromal phase of PD or other synucleinopathies.

Pathological α-syn aggregates are also known to appear in the GI tract in PD patients even decades prior to the onset of motor impairments, but multiple IHC-based studies using GI biopsies have produced conflicting results regarding diagnostic accuracy [15,16,17,19,20]. Therefore, it is of interest whether the α-syn RT-QuIC assay that has demonstrated its diagnostic potential in multiple studies [43,44,45,46,49,58] could detect pathological α-syn aggregates in GI biopsies of PD patients, particularly at the prodromal phase. While the application of α-syn RT-QuIC into GI biopsies has not yet, to the best of our knowledge, been reported, our successful results in detecting α-syn seeding activity in the colon of presymptomatic G2-3 mice strongly support the diagnostic potential of this approach. Additionally, biopsy sites within the GI tract of PD patients could be important for successful detection of α-syn aggregates with seeding activity [20,24]. RT-QuIC-based detection and quantitation of α-syn seeding activity in GI biopsies would be useful not only in the early diagnosis of PD and other synucleinopathies, but also in monitoring disease progression and assessing treatment efficacy [48].

## Figures and Tables

**Table 1:** Summary of the α-syn RT-QuIC assay for brains and colons of G2-3 mice.

Age at Death	ID	Detectable Dilution ^(1),(2)^		Comments
		Brain ^(3)^	Colon ^(4)^	
3 months	3M-#1	ND ^(5)^	10^−4^	
	3M-#2	ND	10^−3^	
	3M-#3	ND	10^−3^	
	3M-Con	ND	ND	non-carrier
6 months	6M-#1	ND	10^−5^	
	6M-#2	10^−3^	10^−5^	
	6M-#3	ND	10^−3^	
	6M-Con	ND	ND	non-carrier
12 months	12M-#1	10^−5^	10^−5^	
	12M-#2	10^−8^	10^−5^	
	12M-#3	10^−5^	10^−5^	
	12M-Con	ND	ND	non-carrier

^(1)^ The fluorescence threshold in each plate was in the range of 15,000–25,000 RFU, which was the basis of determining positive RT-QuIC response. ^(2)^ RT-QuIC seeding activity was detected at least twice from 10^−3^ to indicated dilution(s) in each sample. ^(3)^ Brainstem and midbrain areas were used for RT-QuIC analysis. ^(4)^ Distal part of the colon was used for RT-QuIC analysis. ^(5)^ ND = not detectable at 10^−3^ dilution.

## Data Availability

Not applicable.

## References

[1] Maroteaux L., Campanelli J.T., Scheller R.H. (1988). Synuclein: A neuron-specific protein localized to the nucleus and presynaptic nerve terminal. J. Neurosci..

[2] Jakes R., Spillantini M.G., Goedert M. (1994). Identification of two distinct synucleins from human brain. FEBS Lett..

[3] Polymeropoulos M.H., Lavedan C., Leroy E., Ide S.E., Dehejia A., Dutra A., Pike B., Root H., Rubenstein J., Boyer R. (1997). Mutation in the alpha-synuclein gene identified in families with Parkinson’s disease. Science.

[4] Spillantini M.G., Schmidt M.L., Lee V.M., Trojanowski J.Q., Jakes R., Goedert M. (1997). Alpha-synuclein in Lewy bodies. Nature.

[5] Goedert M. (2001). Alpha-synuclein and neurodegenerative diseases. Nat. Rev. Neurosci..

[6] Goedert M., Spillantini M.G., Del Tredici K., Braak H. (2013). 100 years of Lewy pathology. Nat. Rev. Neurol..

[7] Spillantini M.G., Crowther R.A., Jakes R., Hasegawa M., Goedert M. (1998). alpha-Synuclein in filamentous inclusions of Lewy bodies from Parkinson’s disease and dementia with lewy bodies. Proc. Natl. Acad. Sci. USA.

[8] Papp M.I., Kahn J.E., Lantos P.L. (1989). Glial cytoplasmic inclusions in the CNS of patients with multiple system atrophy (striatonigral degeneration, olivopontocerebellar atrophy and Shy-Drager syndrome). J. Neurol. Sci..

[9] Tu P.H., Galvin J.E., Baba M., Giasson B., Tomita T., Leight S., Nakajo S., Iwatsubo T., Trojanowski J.Q., Lee V.M. (1998). Glial cytoplasmic inclusions in white matter oligodendrocytes of multiple system atrophy brains contain insoluble alpha-synuclein. Ann. Neurol..

[10] Beach T.G., Adler C.H., Sue L.I., Vedders L., Lue L., White Iii C.L., Akiyama H., Caviness J.N., Shill H.A., Sabbagh M.N. (2010). Multi-organ distribution of phosphorylated alpha-synuclein histopathology in subjects with Lewy body disorders. Acta Neuropathol..

[11] Gelpi E., Navarro-Otano J., Tolosa E., Gaig C., Compta Y., Rey M.J., Marti M.J., Hernandez I., Valldeoriola F., Rene R. (2014). Multiple organ involvement by alpha-synuclein pathology in Lewy body disorders. Mov. Disord..

[12] Miki Y., Mori F., Wakabayashi K., Kuroda N., Orimo S. (2009). Incidental Lewy body disease restricted to the heart and stellate ganglia. Mov. Disord..

[13] Fumimura Y., Ikemura M., Saito Y., Sengoku R., Kanemaru K., Sawabe M., Arai T., Ito G., Iwatsubo T., Fukayama M. (2007). Analysis of the adrenal gland is useful for evaluating pathology of the peripheral autonomic nervous system in lewy body disease. J. Neuropathol. Exp. Neurol..

[14] Stokholm M.G., Danielsen E.H., Hamilton-Dutoit S.J., Borghammer P. (2016). Pathological alpha-synuclein in gastrointestinal tissues from prodromal Parkinson disease patients. Ann. Neurol..

[15] Shannon K.M., Keshavarzian A., Mutlu E., Dodiya H.B., Daian D., Jaglin J.A., Kordower J.H. (2012). Alpha-synuclein in colonic submucosa in early untreated Parkinson’s disease. Mov. Disord..

[16] Sanchez-Ferro A., Rabano A., Catalan M.J., Rodriguez-Valcarcel F.C., Fernandez Diez S., Herreros-Rodriguez J., Garcia-Cobos E., Alvarez-Santullano M.M., Lopez-Manzanares L., Mosqueira A.J. (2015). In vivo gastric detection of alpha-synuclein inclusions in Parkinson’s disease. Mov. Disord..

[17] Chung S.J., Kim J., Lee H.J., Ryu H.S., Kim K., Lee J.H., Jung K.W., Kim M.J., Kim M.J., Kim Y.J. (2016). Alpha-synuclein in gastric and colonic mucosa in Parkinson’s disease: Limited role as a biomarker. Mov. Disord..

[18] Ruffmann C., Bengoa-Vergniory N., Poggiolini I., Ritchie D., Hu M.T., Alegre-Abarrategui J., Parkkinen L. (2018). Detection of alpha-synuclein conformational variants from gastro-intestinal biopsy tissue as a potential biomarker for Parkinson’s disease. Neuropathol. Appl. Neurobiol..

[19] Visanji N.P., Marras C., Kern D.S., Al Dakheel A., Gao A., Liu L.W., Lang A.E., Hazrati L.N. (2015). Colonic mucosal a-synuclein lacks specificity as a biomarker for Parkinson disease. Neurology.

[20] Shin C., Park S.H., Yun J.Y., Shin J.H., Yang H.K., Lee H.J., Kong S.H., Suh Y.S., Shen G., Kim Y. (2017). Fundamental limit of alpha-synuclein pathology in gastrointestinal biopsy as a pathologic biomarker of Parkinson’s disease: Comparison with surgical specimens. Parkinsonism. Relat. Disord..

[21] Corbille A.G., Letournel F., Kordower J.H., Lee J., Shanes E., Neunlist M., Munoz D.G., Derkinderen P., Beach T.G. (2016). Evaluation of alpha-synuclein immunohistochemical methods for the detection of Lewy-type synucleinopathy in gastrointestinal biopsies. Acta Neuropathol. Commun..

[22] Beach T.G., Corbille A.G., Letournel F., Kordower J.H., Kremer T., Munoz D.G., Intorcia A., Hentz J., Adler C.H., Sue L.I. (2016). Multicenter Assessment of Immunohistochemical Methods for Pathological Alpha-Synuclein in Sigmoid Colon of Autopsied Parkinson’s Disease and Control Subjects. J. Parkinsons. Dis..

[23] Shin C., Park S.H., Yun J.Y., Shin J.H., Yang H.K., Lee H.J., Kong S.H., Suh Y.S., Kim H.J., Jeon B. (2018). Alpha-synuclein staining in non-neural structures of the gastrointestinal tract is non-specific in Parkinson disease. Parkinsonism. Relat. Disord..

[24] Fenyi A., Leclair-Visonneau L., Clairembault T., Coron E., Neunlist M., Melki R., Derkinderen P., Bousset L. (2019). Detection of alpha-synuclein aggregates in gastrointestinal biopsies by protein misfolding cyclic amplification. Neurobiol. Dis..

[25] Atarashi R., Satoh K., Sano K., Fuse T., Yamaguchi N., Ishibashi D., Matsubara T., Nakagaki T., Yamanaka H., Shirabe S. (2011). Ultrasensitive human prion detection in cerebrospinal fluid by real-time quaking-induced conversion. Nat. Med..

[26] Wilham J.M., Orru C.D., Bessen R.A., Atarashi R., Sano K., Race B., Meade-White K.D., Taubner L.M., Timmes A., Caughey B. (2010). Rapid end-point quantitation of prion seeding activity with sensitivity comparable to bioassays. PLoS Pathog..

[27] Orru C.D., Bongianni M., Tonoli G., Ferrari S., Hughson A.G., Groveman B.R., Fiorini M., Pocchiari M., Monaco S., Caughey B. (2014). A test for Creutzfeldt-Jakob disease using nasal brushings. N. Engl. J. Med..

[28] McGuire L.I., Peden A.H., Orru C.D., Wilham J.M., Appleford N.E., Mallinson G., Andrews M., Head M.W., Caughey B., Will R.G. (2012). Real time quaking-induced conversion analysis of cerebrospinal fluid in sporadic Creutzfeldt-Jakob disease. Ann. Neurol..

[29] Orru C.D., Hughson A.G., Race B., Raymond G.J., Caughey B. (2012). Time course of prion seeding activity in cerebrospinal fluid of scrapie-infected hamsters after intratongue and intracerebral inoculations. J. Clin. Microbiol..

[30] Bongianni M., Orru C., Groveman B.R., Sacchetto L., Fiorini M., Tonoli G., Triva G., Capaldi S., Testi S., Ferrari S. (2017). Diagnosis of Human Prion Disease Using Real-Time Quaking-Induced Conversion Testing of Olfactory Mucosa and Cerebrospinal Fluid Samples. JAMA Neurol..

[31] Henderson D.M., Manca M., Haley N.J., Denkers N.D., Nalls A.V., Mathiason C.K., Caughey B., Hoover E.A. (2013). Rapid antemortem detection of CWD prions in deer saliva. PLoS ONE.

[32] Orrú C.D., Yuan J., Appleby B.S., Li B., Li Y., Winner D., Wang Z., Zhan Y.A., Rodgers M., Rarick J. (2017). Prion seeding activity and infectivity in skin samples from patients with sporadic Creutzfeldt-Jakob disease. Sci. Transl. Med..

[33] Favole A., Mazza M., Vallino Costassa E., D’Angelo A., Lombardi G., Marconi P., Crociara P., Berrone E., Gallo M., Palmitessa C. (2019). Early and Pre-Clinical Detection of Prion Seeding Activity in Cerebrospinal Fluid of Goats using Real-Time Quaking-Induced Conversion Assay. Sci. Rep..

[34] Masujin K., Orru C.D., Miyazawa K., Groveman B.R., Raymond L.D., Hughson A.G., Caughey B. (2016). Detection of Atypical H-Type Bovine Spongiform Encephalopathy and Discrimination of Bovine Prion Strains by Real-Time Quaking-Induced Conversion. J. Clin. Microbiol..

[35] Cramm M., Schmitz M., Karch A., Mitrova E., Kuhn F., Schroeder B., Raeber A., Varges D., Kim Y.S., Satoh K. (2016). Stability and Reproducibility Underscore Utility of RT-QuIC for Diagnosis of Creutzfeldt-Jakob Disease. Mol. Neurobiol..

[36] McGuire L.I., Poleggi A., Poggiolini I., Suardi S., Grznarova K., Shi S., de Vil B., Sarros S., Satoh K., Cheng K. (2016). Cerebrospinal fluid real-time quaking-induced conversion is a robust and reliable test for sporadic creutzfeldt-jakob disease: An international study. Ann. Neurol..

[37] Volpicelli-Daley L.A., Luk K.C., Patel T.P., Tanik S.A., Riddle D.M., Stieber A., Meaney D.F., Trojanowski J.Q., Lee V.M. (2011). Exogenous alpha-synuclein fibrils induce Lewy body pathology leading to synaptic dysfunction and neuron death. Neuron.

[38] Luk K.C., Kehm V., Carroll J., Zhang B., O’Brien P., Trojanowski J.Q., Lee V.M. (2012). Pathological alpha-synuclein transmission initiates Parkinson-like neurodegeneration in nontransgenic mice. Science.

[39] Watts J.C., Giles K., Oehler A., Middleton L., Dexter D.T., Gentleman S.M., DeArmond S.J., Prusiner S.B. (2013). Transmission of multiple system atrophy prions to transgenic mice. Proc. Natl. Acad. Sci. USA.

[40] Woerman A.L., Stöhr J., Aoyagi A., Rampersaud R., Krejciova Z., Watts J.C., Ohyama T., Patel S., Widjaja K., Oehler A. (2015). Propagation of prions causing synucleinopathies in cultured cells. Proc. Natl. Acad. Sci. USA.

[41] Fairfoul G., McGuire L.I., Pal S., Ironside J.W., Neumann J., Christie S., Joachim C., Esiri M., Evetts S.G., Rolinski M. (2016). Alpha-synuclein RT-QuIC in the CSF of patients with alpha-synucleinopathies. Ann. Clin. Transl. Neurol..

[42] Groveman B.R., Orru C.D., Hughson A.G., Raymond L.D., Zanusso G., Ghetti B., Campbell K.J., Safar J., Galasko D., Caughey B. (2018). Rapid and ultra-sensitive quantitation of disease-associated alpha-synuclein seeds in brain and cerebrospinal fluid by alphaSyn RT-QuIC. Acta Neuropathol. Commun..

[43] Iranzo A., Fairfoul G., Ayudhaya A.C.N., Serradell M., Gelpi E., Vilaseca I., Sanchez-Valle R., Gaig C., Santamaria J., Tolosa E. (2021). Detection of alpha-synuclein in CSF by RT-QuIC in patients with isolated rapid-eye-movement sleep behaviour disorder: A longitudinal observational study. Lancet Neurol..

[44] Kang U.J., Boehme A.K., Fairfoul G., Shahnawaz M., Ma T.C., Hutten S.J., Green A., Soto C. (2019). Comparative study of cerebrospinal fluid alpha-synuclein seeding aggregation assays for diagnosis of Parkinson’s disease. Mov. Disord..

[45] Rossi M., Candelise N., Baiardi S., Capellari S., Giannini G., Orru C.D., Antelmi E., Mammana A., Hughson A.G., Calandra-Buonaura G. (2020). Ultrasensitive RT-QuIC assay with high sensitivity and specificity for Lewy body-associated synucleinopathies. Acta Neuropathol..

[46] Bongianni M., Ladogana A., Capaldi S., Klotz S., Baiardi S., Cagnin A., Perra D., Fiorini M., Poleggi A., Legname G. (2019). alpha-Synuclein RT-QuIC assay in cerebrospinal fluid of patients with dementia with Lewy bodies. Ann. Clin. Transl. Neurol..

[47] De Luca C.M.G., Elia A.E., Portaleone S.M., Cazzaniga F.A., Rossi M., Bistaffa E., De Cecco E., Narkiewicz J., Salzano G., Carletta O. (2019). Efficient RT-QuIC seeding activity for alpha-synuclein in olfactory mucosa samples of patients with Parkinson’s disease and multiple system atrophy. Transl. Neurodegener..

[48] Manne S., Kondru N., Jin H., Serrano G.E., Anantharam V., Kanthasamy A., Adler C.H., Beach T.G., Kanthasamy A.G. (2020). Blinded RT-QuIC Analysis of alpha-Synuclein Biomarker in Skin Tissue From Parkinson’s Disease Patients. Mov. Disord..

[49] Manne S., Kondru N., Jin H., Anantharam V., Huang X., Kanthasamy A., Kanthasamy A.G. (2020). alpha-Synuclein real-time quaking-induced conversion in the submandibular glands of Parkinson’s disease patients. Mov. Disord..

[50] Han J.Y., Jang H.S., Green A.J.E., Choi Y.P. (2020). RT-QuIC-based detection of alpha-synuclein seeding activity in brains of dementia with Lewy Body patients and of a transgenic mouse model of synucleinopathy. Prion.

[51] Lee M.K., Stirling W., Xu Y., Xu X., Qui D., Mandir A.S., Dawson T.M., Copeland N.G., Jenkins N.A., Price D.L. (2002). Human alpha-synuclein-harboring familial Parkinson’s disease-linked Ala-53 –> Thr mutation causes neurodegenerative disease with alpha-synuclein aggregation in transgenic mice. Proc. Natl. Acad. Sci. USA.

[52] Yun S.P., Kam T.I., Panicker N., Kim S., Oh Y., Park J.S., Kwon S.H., Park Y.J., Karuppagounder S.S., Park H. (2018). Block of A1 astrocyte conversion by microglia is neuroprotective in models of Parkinson’s disease. Nat. Med..

[53] Martin L.J., Pan Y., Price A.C., Sterling W., Copeland N.G., Jenkins N.A., Price D.L., Lee M.K. (2006). Parkinson’s disease alpha-synuclein transgenic mice develop neuronal mitochondrial degeneration and cell death. J. Neurosci..

[54] Davenport K.A., Hoover C.E., Denkers N.D., Mathiason C.K., Hoover E.A. (2018). Modified Protein Misfolding Cyclic Amplification Overcomes Real-Time Quaking-Induced Conversion Assay Inhibitors in Deer Saliva To Detect Chronic Wasting Disease Prions. J. Clin. Microbiol..

[55] Hoover C.E., Davenport K.A., Henderson D.M., Zabel M.D., Hoover E.A. (2017). Endogenous Brain Lipids Inhibit Prion Amyloid Formation In Vitro. J. Virol..

[56] Brahmachari S., Ge P., Lee S.H., Kim D., Karuppagounder S.S., Kumar M., Mao X., Shin J.H., Lee Y., Pletnikova O. (2016). Activation of tyrosine kinase c-Abl contributes to alpha-synuclein-induced neurodegeneration. J. Clin. Investig..

[57] Rota L., Pellegrini C., Benvenuti L., Antonioli L., Fornai M., Blandizzi C., Cattaneo A., Colla E. (2019). Constipation, deficit in colon contractions and alpha-synuclein inclusions within the colon precede motor abnormalities and neurodegeneration in the central nervous system in a mouse model of alpha-synucleinopathy. Transl. Neurodegener..

[58] Wang Z., Becker K., Donadio V., Siedlak S., Yuan J., Rezaee M., Incensi A., Kuzkina A., Orru C.D., Tatsuoka C. (2020). Skin alpha-Synuclein Aggregation Seeding Activity as a Novel Biomarker for Parkinson Disease. JAMA Neurol..

